# *Cornus mas* (Linnaeus) Novel Devised Medicinal Preparations: Bactericidal Effect against *Staphylococcus aureus* and *Pseudomonas aeruginosa*

**DOI:** 10.3390/molecules200611202

**Published:** 2015-06-17

**Authors:** Anthony M. Kyriakopoulos, Biswanath Dinda

**Affiliations:** 1 Nasco AD Biotechnology Laboratory, 11 Sachtouri Street, 18536 Pireus, Greece; 2 Department of Chemistry, Tripura University, Suryamaninagar 799 022 Tripura, India; E-Mail: dindabtu@gmail.com

**Keywords:** *Cornus mas* (L.) fresh fruits, novel medicinal preparations, selective bactericidal effect, *Pseudomonas* aeruginosa, *Staphylococcus aureus*

## Abstract

The medicinal properties of *Cornus mas* L. (=*Cornus mascula* L.), Cornaceae, are well described in Hippocratian documents, and recent research provides experimental evidence for some of these properties. However, the chemical components of *Cornus mas* L. that may be of pharmaceutical importance are relatively unstable. In this respect a novel methodology for plant nutrient element extraction that provides favorable conditions for simultaneous stabilization of such fragile and unstable structures has been devised. Using this methodology, medicinal preparations derived from *Cornus mas* L. fresh fruits, proved to possess significant antimicrobial activity selective against *S. aureus* and *P. aeruginosa*. This effect became apparent with the addition of sodium bromide in the extraction procedure and varied with the ion availability during extraction. The identification of novel agents with potent antimicrobial activity against these species is of medical importance to overcome the problem of universal antibiotic resistance.

## 1. Introduction

Studies on species of the genus *Cornaceae*, provide data of pharmaceutical importance. Hypoglycaemic activity with anti-diabetic potential was recorded for total iridoid glycoside methanol extracts from *Cornus officinalis* [1], and anti-hyperglycemic together with anti-hyperlipidemic effects, were recorded for methanol extracts of *Cornus mas* L. [2]. The cytoprotective effects of methanol-water extracts of *Cornus mas* L. fruits, were recorded in an induced hepatotoxicity, animal model [3]. Moreover, other data [4,5] support traditional medicine uses that describe antimicrobial, antiparasitic, and anti-allergic properties in *Cornus mas* L. extracts. Fresh fruits of *Cornus mas* L., produced in Northern Greece exhibit high antioxidant activity when measured as ascorbic acid equivalents and compared to other fruits produced at the same location [6]. The extraction solvents methanol and ethanol, depending on their aqueous percentage composition, have different effects on the total extraction yield measured as the total phenolic and the flavonoid concentration that reflects the reducing power due to the antioxidant components extracted from the plant material, amongst other measurable properties [7]. Complementary, other studies suggest that the extraction of the endogenous plant elements depends on the efficiency of the extraction method used [8,9]. Moreover, it has been shown that by following an acidic extraction method, the stability of *Cornus mas* L. elements is limited; anthocyanines, the glucosides of anthrocyanidines and major pigments of these fruits, are both pH and heat sensitive, unless stored at 2 °C [10]. Also, their physicochemical characteristics are altered at room temperature, even when food preservatives are added to their aqueous solutions [11]. In this study we focused to devise more efficient ways of extraction protocols for plant material of medicinal importance. In this respect we have developed a novel aqueous extraction methodology, namely, the “Aqueous Acidic Preparation used for soft Alkaline Lysis, Extraction and Biochemical Treatment of the *Cornus mascula* Fresh Fruit Nutritional Elements” [12]. Anti-cancer activity obtained using this methodology is in process of being patented. This methodology was devised in order to biochemically stabilize, modify, and simultaneously preserve the fragile nutrient structures of these particular *Cornus mas* L. fresh fruits, harvested from the wild and unpolluted forests of Northern Greece. Using this methodology, a particular medicinal preparation showed an even stronger and direct, with no reversal of viability, bactericidal effect, on *Staphylococcus aureus* and *Pseudomonas aeruginosa* [13]. Results were obtained through the antimicrobial preservative effectiveness test commonly used for antimicrobial/preservative activity for approval of products directed to the cosmeceutical and pharmaceutical industry [14]. The tested solutions are novel medicinal preparations, produced by a patent device/methodology of production. These pioneering, biochemically modified aqueous extracts from *Cornus mas* L. fruits, show an indisputable bactericidal activity, which emerges due to the presence of sodium bromide. Their potency is differentiated by the days of bacterial incubation needed to achieve total killing of *S. aureus* and *P. aeruginosa*. The only experimental difference between procedures is the ion availability during extraction. The aim of this study was to provide novel extraction procedures that may lead to plant derived preparations of medical importance.

## 2. Results and Discussion

### 2.1. Cornus mas L. Medicinal Preparations

**Figure 1:**
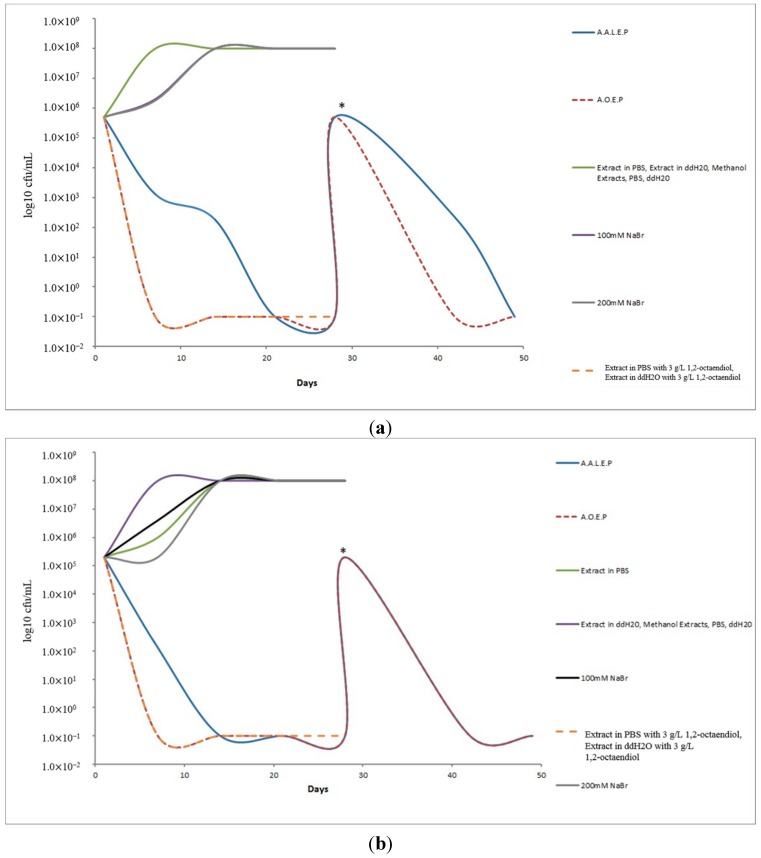

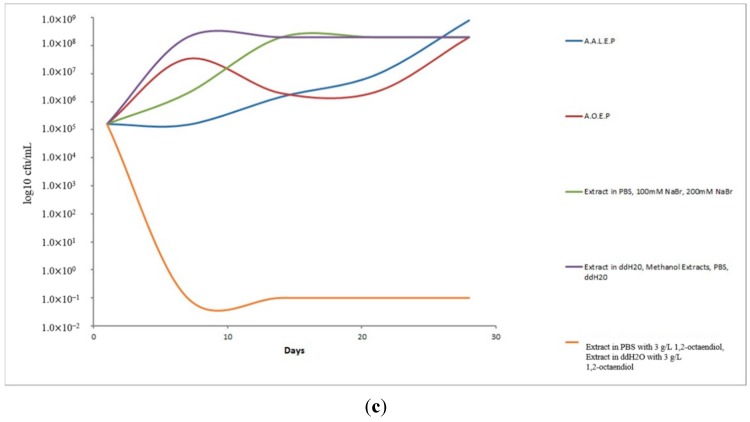
Antibacterial effects of *Cornus mas* L. extracts against (**a**) *S. aureus*; (**b**) *P. aeruginosa*. (**c**) *C. albicans*. Both A.A.L.E.P. and A.O.E.P. preparations exert bactericidal activity against *S. aureus* and *P. aeruginosa* whereas growth of *C. albicans* was not affected. * The effects were still evident after second inoculation on day 28 when the A.A.L.E.P. and A.O.E.P. test vials were inoculated again with same count of *S. aureus* (**a**) and *P. aeruginosa* (**b**) as day 1, to test the duration and strenth of the activity. Experiments were repeated three different times and figures were drawn from mean value results from all three experiments (for Standard Deviations see Table 1, Table 2 and Table 3).

Using novel methodologies for nutrient extraction from the fresh flesh *of Cornus mas* L. fruits that focus on providing the appropriate conditions to maintain the biological activity of their fragile structures, we have obtained preparations with potent bactericidal activity against *S. aureus* and *P. aeruginosa* (Figure 1a,b, Table 1 and Table 2) as compared to PBS and aqueous extraction preparations.

**Table 1:** Bactericidal activity of different *Cornus mas* L. extracts against *S. aureus* (ATCC6538).

Tested Solution	Initial Inoculum cfu/mL	Day 7 Average ^a^ Value (SD ^b^)	Day 14 Average Value (SD)	Day 21	Day 28	SecondInoculum cfu/mL ^c^	Day 42 Average Value (SD)	Day 49
A.A.L.E.P	5 × 10^5^	1.3 × 10^3^ (2 × 10^2^)	1.8 × 10^2^ (3 × 10)	<10	<10	5 × 10^5^	2.2 × 10^2^ (40)	<10
A.O.E.P	5 × 10^5^	<10	<10	<10	<10	5 × 10^5^	<10	<10
Extract in PBS	5 × 10^5^	>10^7^	NMC ^d^	NMC	NMC	-	-	-
Extract in ddH_2_O	5 × 10^5^	NMC	NMC	NMC	NMC	-	-	-
Methanol Extracts	5 × 10^5^	NMC	NMC	NMC	NMC	-	-	--
PBS/ddH_2_O	5 × 10^5^	NMC	NMC	NMC	NMC	-	-	-
100 mM NaBr	5 × 10^5^	2 × 10^6^	>10^8^	NMC	NMC	-	-	-
200 mM NaBr	5 × 10^5^	1.8 × 10^6^	>10^8^	NMC	NMC	-	-	-
Extract in PBS & Extract in ddH_2_O + 3 g/L octanediol	5 × 10^5^	<10	<10	<10	<10	-	-	-

^a^ These are the mean average values of results obtained by three different experiments; ^b^ SD: Standard Deviation calculated by formula: $s=\sqrt{\frac{\sum {\left(X-\bar{X}\right)}^{2}}{n-1}}$; ^c^ At day 28, to check the endurance of antimicrobial activity of A.A.L.E.P. & A.O.E.P. the experiment was continued by repeating the inoculation of *S. aureus* only for these preparations; ^d^ NMC: not measurable colonies, >10^8^ cfu/mL.

**Table 2:** Bactericidal activity of different *Cornus mas* L. extracts against *P. aeruginosa*.

Tested Solution	Initial Inoculum cfu/mL	Day 7 Average ^a^ Value (SD ^b^ )	Day 14	Day 21	Day 28	SecondInoculumcfu/mL ^c^	Day 42	Day 49
A.A.L.E.P	2 × 10^5^	1.3 × 10^2^ (3× 10)	<10	<10	<10	2 × 10^5^	<10	<10
A.O.E.P	2 × 10^5^	<10	<10	<10	<10	2 × 10^5^	<10	<10
Extract in PBS	2 × 10^5^	>10^6^	NMC	NMC	NMC	-	-	-
Extract in ddH_2_O	2 × 10^5^	NMC ^d^	NMC	NMC	NMC	-	-	-
Methanol Extracts	2 × 10^5^	NMC	NMC	NMC	NMC	-	-	-
PBS/ddH_2_O	2 × 10^5^	NMC	NMC	NMC	NMC	-	-	-
100 mMNaBr	2 × 10^5^	4 ×10^6^ (2 × 10^6^)	NMC	NMC	NMC	-	-	-
200 mM NaBr	2 × 10^5^	2 × 10^5^ (2 × 10^5^)	NMC	NMC	NMC	-	-	-
Extract in PBS & Extract in ddH_2_O + 3 g/L octanediol	2 × 10^5^	<10	<10	<10	<10	-	-	-

^a^ Mean average values of results obtained by three different experiments; ^b^ SD: Standard Deviation; ^c^ At day 28, to check the endurance of antimicrobial activity of A.A.L.E.P. and A.O.E.P. the experiment was continued by repeating the inoculation of *P. aeruginosa* only for these preparations.; ^d^ NMC: not measurable colonies: >10^8^ cfu/mL.

**Figure 2:**
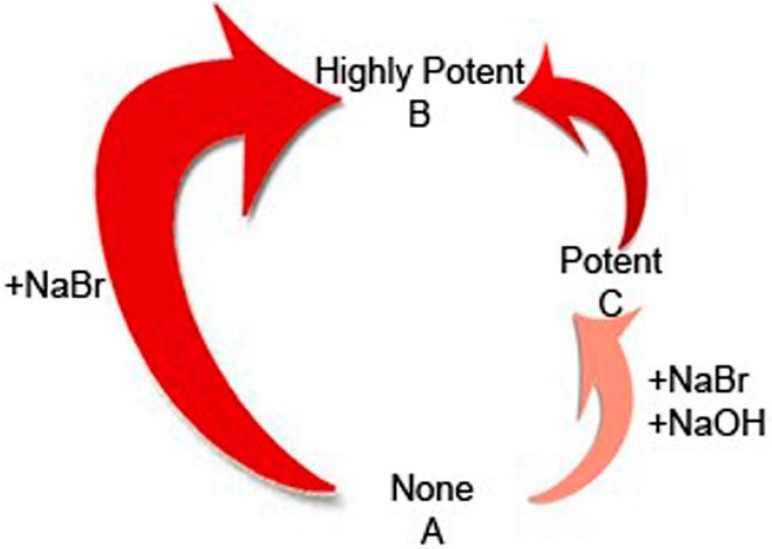
Bactericidal effect on *S. aureus* and *P. aeruginosa*.

Using a well-controlled series of experiments (Experimental section), we have isolated as a sole contributing factor for obtaining this potent bactericidal activity of the preparations, the addition of 200 mM sodium bromide to the flesh of fresh *Cornus mas* L. fruits at 4 °C, for 24 h. Trying to evaluate the reaction of sodium bromide salts with the constituents of the fresh fruits, we have noticed that the introduction of a reducing agent, specifically 10 mM of NaOH to the pH of 200 mM sodium bromide to a value of 10, transiently inhibited the bactericidal activity for both *S. aureus* and *P. aeruginosa* (Figure 1a,b and Figure 2).

#### 2.1.1. Aqueous Alkaline Lysis Extraction Procedure (A.A.L.E.P)

The A.A.L.E.P. is a patented methodology for producing a medicinal preparation [12]. With this preparation, *S. aureus* microbial count seemed to decrease by 2.388 × 10^3^ cfu/mL/day (average value from three experiments conducted separately), SD:1, until bacterial killing was noticed at the 21st day of incubation. More evidently, *P. aeruginosa* seemed to decrease in microbial count by 14.295 × 10^3^ cfu/mL/day, (average value) SD:2, until bacterial killing was noticed at the 14 day of incubation (Figure 1a,b, Table 1 and Table 2).

#### 2.1.2. Aqueous Osmotic Extraction Procedure (A.O.E.P)

The A.O.E.P method [13] resulted in yet another medicinal preparation which killed bacteria in less time than the A.A.L.E.P. preparation. In this procedure, no alkaline lysis of the fresh fruit tissue structures was involved, and instead nutritional elements were extracted by osmosis due to the higher external concentration of NaBr. This preparation killed bacteria in less time (noticed at the 7th day of incubation) and the decrease in microbial count for *S. aureus* was 71,428 × 10^3^ cfu/mL/day and for *P. aeruginosa* 28,572 × 10^3^ cfu/mL/day (the same for all three experiments, Figure 1a,b, Table 1 and Table 2).

### 2.2. Bactericidal Effect

The A.O.E.P medicinal preparation had a rapid antibacterial activity leading to a bactericidal effect at the 7th day checkpoint which remained unchanged until the 28th day interval of recording (Figure 1a,b). The antibacterial activity for the A.A.I.E.P. preparation was substantial at the 7th day checkpoint and continued over to end up to a bactericidal effect on *S. aureus* strains at the 21st day and on *P. aeruginosa* strains at the 14th day, as no bacterial growth was observed until the 28th day of incubation (Figure 1a,b). Moreover, the same preparations A.A.L.E.P, and A.O.E.P., after bacterial killing was recorded at the 28th day, where inoculated again with equal count of *S. aureus* and *P. aeruginosa*, respectively. A remarkable stability in their bactericidal activity for a post-14 day period was recorded again (Figure 1a,b). The bactericidal effect against *S. aureus* and *P. aeruginosa* due to the addition of 200 mM NaBr during extraction (amongst *Cornus mas* L. extracts tested in this study, *n* = 7) was statistically significant: *Chi Square Value:* 1679 ((df = 1, *p* = 0.2) = 1.642). The addition of 10 mM NaOH, transiently reduced this bactericidal effect (Figure 1a,b).

### 2.3. Cornus mas L. Fruit Nutritional Elements

The pharmaceutical importance of *Cornus mas* L. nutritional elements was discussed recently [2,3]. Glycosides and ascorbic acid equivalent-like structures are the major molecules of the fruits of *Cornus mas* L. [6,15]. The significant total iridoid glycoside content with anti-diabetic properties of another species of Cornaceae, *Cornus officinalis*, has also been described [1]. A phytochemical investigation of the composition of the particular *Cornus mas* L. fruits used in this study has been performed by our team [16]. From the nine compounds identified, four were flavonoids, two had acidic structures, one was d-glucose and the other two were novel structures (one flavonoid and one chalcone structure). The flavonoid structures are known to naturally exist in their glycoside forms [17,18,19,20]. The acidic structures identified, namely ursolic acid and gallic acid, may be responsible for the acidic (pH < 5) nature of the extracts. The results from the antimicrobial effectiveness test, obtained by the A.A.L.E.P and A.O.E.P extraction protocols and their respective novel medicinal preparations, clearly show them as having potent antimicrobial activities as compared to the methanol, PBS and aqueous extraction preparations (Figure 1a,b). By using control experiments, it has been shown that it is the introduction of sodium bromide salt in the extraction buffered solutions in PBS, either at pH 7.2 or titrated to pH 10 with NaOH, that caused this effect, as even the 200 mM NaBr solution in PBS had no bactericidal activity (Figure 1a,b, Table 1 and Table 2). The methanol extract, mimicking the ddH_2_O and PBS extraction preparations, possessed limited, if any bactericidal activity that could not be measured in our tests (Figure 1a,b, Table 1 and Table 2). This finding is supported by studies showing that organic solvent extraction has several disadvantages compared to ionic liquid extraction [21] and that certain halide aqueous solutions form strong and stable molecular structures by hydrogen bonding in ionic liquids [22].

Ionic liquids can be compared to ionic solutions, as is in this case the sodium bromide solution of 200 mM, used for extraction, (melting temperature at 755 °C), that contain both sodium and bromide ions together with neutral molecules [23]. 

#### Favorable Conditions by the Novel Methodology to Generate Stable Compounds

**Scheme 1:**
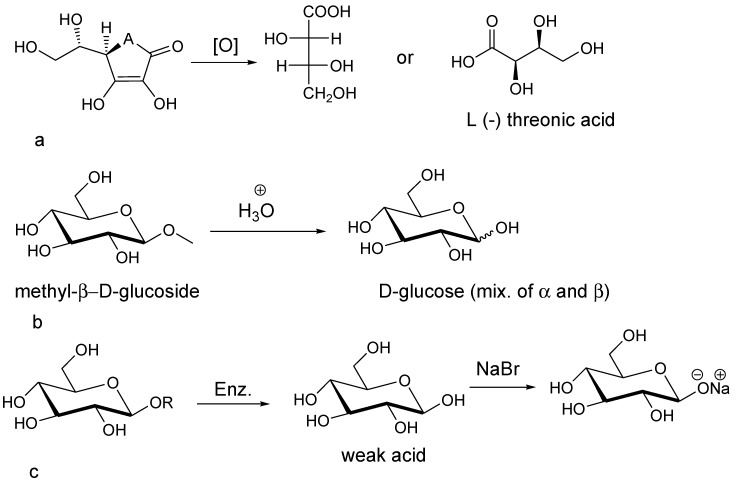
(**a**) Degradation of l-(+)-ascorbic acid to l-(−)-threonic acid, (**b**) Degradation of d-glycosides to d-glucose and (**c**) Salt formation of glycoside structures.

The novel methodology introduced for extracting the compounds from these fruits, takes into account and pays particular attention to the stabilization of their fragile and unstable structures [14,24]. For example, molecules like l-(+)-ascorbic acid (Scheme 1a) and d-glucosides (Scheme 1b) may be easily degraded in terms of their natural conformational forms and due to their chemical instability [14,25,26].

Following application of the A.A.L.E.P methodology, molecules like l-(+)-ascorbic acid and d-glycosides in their transient forms as found in the fresh fruits have the opportunity to react and form more stable salt structures (Scheme 1c). The A.O.E.P methodology does not involve an alkaline lysis step (pH 10), like the A.A.L.E.P does. In the fruits of *Cornus mas* L., the major class of chemical constituents are phenolics including flavonoids [16]. In alkaline pH, all these compounds come in to the extract and exhibit their anti microbial activities as enolates (intermediate forms) [27,28].

**Scheme 2:**
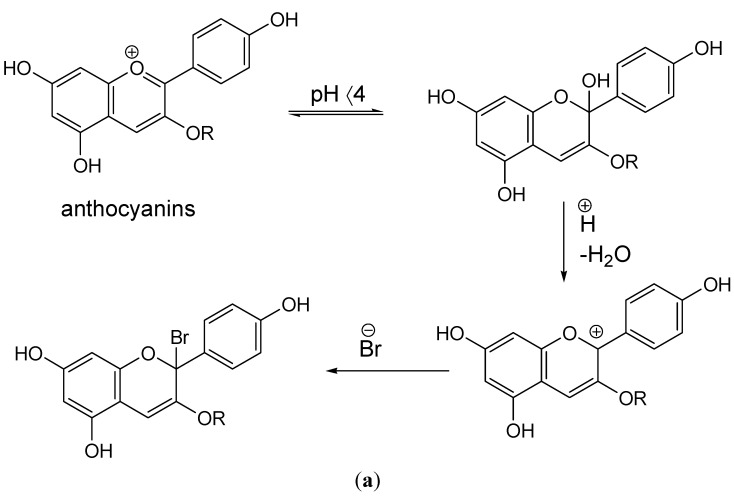

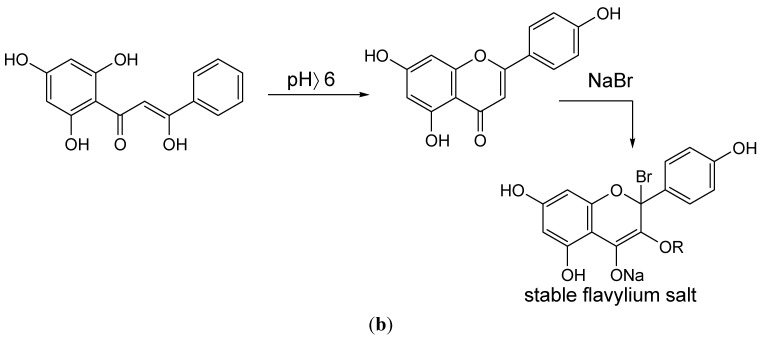
(**a**) Possible formation of stable anthrocyanide-bromide salts at an acidic pH and (**b**) of stable flavylium salts at a basic pH. Basic flavonoid structure via its cationic condition can form stable flavylium salts with bromide.

Anthocyanidins and their glucosides are unstable, easily degraded compounds that are easily depigmented [10]. We note that no depigmentation was observed during our experiments with our novel extraction methiods but this was apparent during our tests with the methanol and other *C.* *mas* extracts. All the flavonoid structures that we have discovered in our previous phytochemical analysis [14], especially when present in an acidic biological environment, can exist in their flavylium cation form [29]. Anthocyanins (Scheme 2a) and chalcones present in the fruits possibly produce stable flavylium salts (Scheme 2b) depending on pH (A.A.L.E.P and A.O.E.P, medicinal preparations). These flavylium salts possess key roles in bactericidal activity [30]. The introduction of a reducing agent, like sodium thiosulphate to the bactericidal extracts on future investigations, will help to clarify these antimicrobial properties. On these grounds, important biological properties of 1-benzopyrans have been described [31], and moreover, reactions with flavonoid structures and bromide compounds have also been scarcely described [32,33]. It should also be noted that together with the supplementation of 200 mM NaBr to the extraction mixture, PBS pH 7.2 used so all our experiments had final concentrations of 136, 88 mM NaCl, 2.68 mM KCl, 12 mM Na_2_HPO_4_, 1.72 mM KH_2_PO_4._ These oxygen and chloride-containing reactants create favorable conditions, especially during the alkaline lysis extraction, of generating reactive halo compounds (ΟBr^−^, OCl^−^) [34]. These may exert highly potent bacteriocidal activity, although this has only been proved for mammalian tissue so far [35,36,37,38]. Nevertheless, by focusing on the scope of this study, as the results show clearly, we have proved that the interaction of sodium bromide and plant tissue as a sole factor during extraction, leads to final preparations with bactericidal properties and that this activity is somehow transiently inhibited when the extraction is being carried out at an alkaline environment (10 mM NaOH presence, Figure 2).

### 2.4. Selective Antimicrobial Inhibition

Interestingly, the same medicinal preparations that show potent antimicrobial activity, fail to inhibit the growth of eukaryotic cells of *Candida albicans*. The results of *C. albicans* growth, obtained by the respected medicinal preparations indicate a selective antibacterial activity, causing a prokaryotic rather than a eukaryotic toxic effect (Figure 1a–c, Table 3). The vast difference between prokaryotic and eukaryotic chromosomes direct for such a distinct structural organization that, this only may reflect the diversity of antibacterial and antifungal agents [39,40]. Moreover, it can be said that the mode of selective antibacterial activity of the respected novel medicinal preparations seems to be independent of the common mechanisms of cytotoxicity between bacteria and fungi [41]. Since both A.A.L.E.P and A.O.E.P. derived medicinal preparations (Figure 1a,b and Figure 2) are bactericidal against both *S. aureus* and *P. aeruginosa* strains, it can be said that this activity is independent of the bacterial cell wall structural differences between Gram positive and negative bacteria. Common antimicrobial activity across Gram positive and negative species is seen with the β-lactams [42], quinolones [43], rifampicin and other antibacterial agents [44].

#### Relevance to the Antimicrobial Resistance Trends of *S. aureus* and *P. aeruginosa*

Antibiotic resistance noticed at the early years of penicillin clinical use [45] has progressed over the years [46,47], sounding nowadays a global alarm for *S. aureus* and *P. aeruginosa* infections [48,49,50,51,52,53,54]. Complementarily, their capability of biofilm formation makes these species almost impossible to eradicate [55,56]. Recent studies are trying to find new antimicrobials to combat multi-drug resistance [57,58]. The discovery of novel agents like the A.A.L.E.P and the A.O.E.P. preparations with potent antimicrobial activity against *S. aureus* and *P. aeruginosa*, is considered to be of medical importance due to these facts. The discovery of novel agents like the A.A.L.E.P and the A.O.E.P. preparations with potent antimicrobial activity against *S. aureus* and *P. aeruginosa*, is considered to be of medical importance due to these facts. It is for the first time such data, to be publicized for these particular plant extraction protocols. This report is considered to be the first ever, to describe the antimicrobial activity of *Cornus mas* L. nutritional elements, by using these novel medicinal preparations.

**Table 3:** Effect of *Cornus mas* L. extracts against *Candida albicans* (ATCC 10231).

Tested Solution	Initial Inoculum cfu/mL	Day 7 Average Value ^a^ (SD ^b^)	Day 14 Average Value (SD)	Day 21 Average Value (SD)	Day 28 Average Value (SD)
A.A.L.E.P	1.6 × 10^5^	1.5 × 10^5^ (4 × 10^4^)	1.5 × 10^6^ (5 × 10^5^)	8.9 × 10^6^ (9 × 10^5^)	8 × 10^8^ (9 × 10^7^)
A.O.E.P	1.6 × 10^5^	1.8 × 10^5^ (6 × 10^4^)	2× 10^6^ (8 × 10^5^)	2.2× 10^6^ (8 × 10^5^)	2 × 10^8^ (1.1 × 10^7^)
Extract in PBS	1.6 × 10^5^	2 × 10^6^ (1 × 10^5^)	NMC ^c^	NMC	NMC
Extract in ddH_2_O	1.6 × 10^5^	NMC	NMC	NMC	NMC
Methanol Extracts	1.6 × 10^5^	NMC	NMC	NMC	NMC
PBS/ddH_2_O	1.6 × 10^5^	NMC	NMC	NMC	NMC
100 mM and 200 mM NaBr	1.6 × 10^5^	2 × 10^6^ (1 × 10^5^)	NMC	NMC	NMC
Extract in PBS and Extract in ddH_2_O + 3 g/L octanediol	1.6× 10^5^	<10	<10	<10	<10

^a^ These are the mean average values of results obtained by three different experiments; ^b^ SD: Standard Deviation; ^c^ NMC: not measurable colonies: >10^8^ cfu/mL.

## 3. Experimental Section

### 3.1. Materials

#### 3.1.1. Plant Material

Fresh fruits of *Cornus mas* L. were collected from unpolluted and wild forests of Northern Greece in the season of early autumn 2012 and kept at −20 °C. A specimen was deposited at the Herbarium Depository Laboratory of the National and Kapodistrian University of Athens, identified as *Cornus mas.* L. by Professor Th. Constantinidis, and designated with the No: akyriakopoulos s.n. 5/9/2013.

#### 3.1.2. Extraction Material

Double distilled water was from Carlo Erba Reagents (Bristol, PA, USA), Analytical grade methanol, phosphate buffered saline, sodium bromide and sodium hydroxide were from Sigma Aldrich (St. Louis, MO, USA). Filter separation membranes from Ahlstrom Munktell (Bärenstein, Germany).

#### 3.1.3. Microbiology Material & Equipment

*Stahylococcus aureus* (ATCC No. 6538), *Pseudomonas aeruginosa* (ATCC No. 9027), Soybean Casein Agar and Broth from Sigma Aldrich, Microbiology incubator HERATHERM™ (Thermo Scientific, Waltham, MA, USA), Turdidimeter (HACH, Loveland, CO, USA).

### 3.2. Methods

#### 3.2.1. A.A.L.E.P.

The fresh fruits of *Cornus mas* L., kept at −20 °C immediately post-harvesting, were cleaned thoroughly in ddH_2_O at 5 °C, the fruit flesh was separated manually from the core, and mixed under aseptic conditions with 200 mM NaBr phosphate buffered solution pH 10 NaOH in a 1:1 *w*/*v* proportion. The mixture was kept at 4 °C for 24 h, under slow but continuous stirring motion. The aqueous part of mixture was separated from the solid material with specific 100% cellulose nanomembares, and kept at −20 °C until use.

#### 3.2.2. A.O.E.P.

Exactly the same procedure was followed as with the A.A.L.E.P. methodology, apart from that in the same 1:1 *w*/*v* proportions a solution of 200 mM NaBr in PBS pH 7.2, was used instead of 200 mM NaBr, pH 10 in PBS.

#### 3.2.3. PBS Extraction

Briefly the same procedure as described for A.A.L.E.P. was followed, but in the 1:1 *w*/*v* mixture sterile PBS with no additives was used in the same ddH_2_O. PBS extraction was used to test that it is the addition of NaBr in the A.A.L.E.P. and A.O.E.P. that results in the killing of *S. aureus* and *P. aeruginosa*. 

#### 3.2.4. ddH_2_O Extraction

Briefly, the same procedure was followed as A.A.L.E.P, but in the 1:1 *w*/*v* mixture, sterile ddH_2_O was used instead. This was used to test that it is the addition of NaBr in the A.A.L.E.P. and A.O.E.P. that results to the killing of *S. aureus* and *P. aeruginosa*. 

#### 3.2.5. Methanol Extractions

Equal portions of flesh from fresh *Cornus mas* L. fruits stored at −20 °C, were separated from the core and were mixed with methanol/water (80/20 *v*/*v*) solution, in 1:1 ratio. The mixture was kept at 4 °C for 24 h under a slow but continuous stirring motion. The liquid phase was separated from the solids by a nanomembrane, and the methanol was evaporated on a water bath at 30 °C. The aqueous part was briefly kept at 4 °C prior to testing. 

Same portions of fresh fruits were left to dry at room temperature. The core was separated from the dried flesh, semipowdered, and mixed with the same amount of methanol/water (80/20 *v*/*v*) as for fresh fruits in a 1:1 ratio. The mixture was kept at 4 °C for 24 h under a slow but continuous stirring motion. The liquid phase was separated from the solids by a nanomembrane, and the methanol was evaporated on a water bath at 30 °C. The aqueous part was briefly kept at 4 °C prior to testing. 

Both methanol extracts failed to kill *S. aureus* and *P. aeruginosa*.

#### 3.2.6. PBS

Microbial strains of *S. aureus* (ATCC No. 6538) and *P. aeruginosa* were inoculated in sterile PBS only (made up of the same ddH_2_O) for growth testing. Inoculation of bacteria in PBS was used to test that the PBS solution used does not contribute to the killing of *S. aureus* and *P. aeruginosa.*

#### 3.2.7. ddH_2_O

Microbial strains of *S. aureus* (ATCC No. 6538) and *P. aeruginosa* (ATCC No. 9027) were inoculated in sterile ddH_2_O for growth testing. Inoculation of bacteria in ddH_2_O was used to test that ddH_2_O solution used does not contribute to the killing of *S. aureus* and *P. aeruginosa.*

#### 3.2.8. 100 mM NaBr and 200 mM NaBr

Microbial strains of *S. aureus* (ATCC No. 6538) and *P. aeruginosa* (ATCC No. 9027) were inoculated in sterile 100 and 200 mM NaBr solutions, respectively, in PBS (made up of the same ddH_2_O), for growth testing. Inoculation of bacteria in 100 mM and 200 mM NaBr in PBS pH 7.2 was used to test that neither solution contributes to the killing of *S. aureus* and *P. aeruginosa*.

#### 3.2.9. Extract in PBS with 3 g/L 1,2-octanediol and Extract in ddH_2_O with 3 g/L 1,2-octanediol

Preparations from PBS extraction and ddH_2_O extraction were used and made up to 3 g/L with 1,2-octanediol, commonly used as a safe preservative [59]. Inoculation of bacteria and fungi in PBS and ddH_2_O, 3 g/L 1,2-octanediol was used to test whether the addition of a potent preservative in the respective solutions would lead to the killing of *S. aureus*, *P. aeruginosa* and *C. albicans.*

#### 3.2.10. Antimicrobial Preservative Effectiveness Test Category 2

Antimicrobial effectiveness tests were performed according to the standards of the US Pharmacopeia [12] and to the European regulation 1223/2009. Specifically, fresh (not more than five passages from the original stock) inoculating microorganisms in broth were centrifuged, the pellet was resuspended in 1/20th the volume of fresh maintenance broth and an equal volume of 20% of sterile glycerol (*v*/*v*) in water was added. Growth was promoted in Soybean-casein digest broths at 32.5 ± 2.5 °C for 5 days and microbial specimens were separated in aliquots and kept at liquid nitrogen until use. Inocula were prepared from freshly grown cultures of specific *S. aureus* and *P. aeruginosa* strains that were harvested using sterile saline Tryptic Soy Broth from Soybean-casein digest agar plates and measured by dilution to about 1 × 10^8^ cfu/mL by turbidimetric measurement. Five original containers of 20 mL of each tested solution were prepared and inoculated with a volume of 0.1–0.2 mL of microorganisms to obtain 1 × 10^5^ to 1 × 10^6^ cfu/mL for each solution. Prior to inoculation suitable volumes of 0.1–0.2 mL were removed from the test containers. Tested solutions were incubated at 32.5 ± 2.5 °C for 7, 14, 21 and 28 day time intervals and more. At the expiration of each interval, 1 mL of tested solution was tested for microbial growth as cfu/ mL by using series of 10-fold dilutions and the plate method for colony measurement. No microbial growth was designated as <10. Extreme growth was designated as >10^8^. Experiments were repeated for three times. A microbiological sterility test was also performed on all reagents used.

## 4. Conclusions

A.A.L.E.P. and A.O.E.P. medicinal preperations display bactericidal effects on strains of *S. aureus* and *P. aeruginosa* attributed to the presence of 200 mM sodium bromide during extraction and its influence on the naturally occurring content of the *Cornus mas* L. fresh fruits. This bactericidal effect depends on ion availability during extraction as the addition of sodium hydroxide, transiently reduced this effect. Finally this effect appears to be selective for prokaryotes as *C. albicans* was hardly affected. Further research will identify key molecular reactions that lead to this antibacterial activity. 
